# A Case Report of Systemic Allergic Reaction to the Dual Glucose-Dependent Insulinotropic Polypeptide/Glucagon-Like Peptide-1 Receptor Agonist Tirzepatide

**DOI:** 10.7759/cureus.51460

**Published:** 2024-01-01

**Authors:** Trang Thi Bich Le, Le Huu Nhat Minh, Pooja Devi, Nabila Islam, Issac Sachmechi

**Affiliations:** 1 Internal Medicine, Department of Cardiovascular Research, Methodist Hospital, Merrillville, USA; 2 College of Medicine, Taipei Medical University, Taipei, TWN; 3 Research Center for Artificial Intelligence in Medicine, College of Medicine, Taipei Medical University, Taipei, TWN; 4 Internal Medicine, Maimonides Medical Center, New York, USA; 5 Internal Medicine, Mymensingh Medical College, Dhaka, BGD; 6 Internal Medicine, Queens Hospital Center, New York, USA; 7 Internal Medicine, Icahn School of Medicine at Mount Sinai/Queens Hospital Center, New York, USA

**Keywords:** safety, hypersensitivity, allergic reaction, gip/glp-1 receptor agonist, tirzepatide

## Abstract

This report examines a case of systemic hypersensitivity to tirzepatide in a patient with type 2 diabetes. Tirzepatide (Mounjaro®), a dual agonist of glucose-dependent insulinotropic polypeptide (GIP) and glucagon-like peptide-1 (GLP-1) receptor, has recently gained FDA approval. Additionally, a literature review was conducted to summarize recent research on tirzepatide's effectiveness and safety.

A 67-year-old woman, previously treated with basal insulin, metformin, and semaglutide (a GLP-1 agonist), experienced severe disseminated pruritus and a generalized urticarial rash after her first dose of tirzepatide.

This reaction, which subsided with antihistamines, raises questions about possible immunoglobulin E-mediated hypersensitivity. The report highlights the need for increased vigilance regarding allergic reactions to new diabetes medications, particularly in the context of GIP/GLP-1 receptor agonists.

## Introduction

Diabetes and obesity are prevalent chronic diseases that cause a considerable burden of disease and mortality worldwide, particularly in developed countries [1]. In light of this, there is a need for advanced treatment options that can provide better clinical outcomes. Tirzepatide, alternatively identified as Mounjaro®, represents a pioneering pharmaceutical compound functioning as the exclusive dual agonist targeting the glucose-dependent insulinotropic polypeptide (GIP) and glucagon-like peptide-1 (GLP-1) receptors. This innovative medication exhibits notable efficacy in the reduction of blood glucose levels, enhancement of insulin sensitivity, and promotion of body weight reduction. Tirzepatide is administered as a once-weekly subcutaneous injection and was first approved by the US Food and Drug Administration (FDA) for the management of type 2 diabetes mellitus (T2DM) in May 2022 [2]. It is currently undergoing clinical trials for the treatment of heart failure, obesity, cardiovascular diseases in T2DM, and non-alcoholic steatohepatitis. Tirzepatide has demonstrated a dose-dependent effect in lowering glycosylated hemoglobin (HbA1c) levels and reducing body weight, all while maintaining a favorable safety profile without an elevated risk of hypoglycemia. Still, there is an increase in gastrointestinal adverse events like nausea, vomiting, and diarrhea [3]. The adverse drug reaction profile of tirzepatide closely resembles that of GLP-1 agonists, with allergic reactions, particularly potentially life-threatening allergies like anaphylactic reactions, having been reported across most GLP-1 receptor agonists [4]. The incidence of anaphylactic reactions to tirzepatide, a GLP-1 receptor agonist, has been documented but appears to be rare [5]. A case report highlighted a biphasic anaphylactic reaction induced by tirzepatide, indicating that while allergic reactions have been reported during clinical trials, they are not common occurrences. In addition, tirzepatide is a relatively new medication in the market. Therefore, it is crucial to be vigilant for any allergic reactions to ensure its safety and efficacy.

We present a novel case illustrating systemic hypersensitivity reactions associated with the administration of tirzepatide, a GIP/GLP-1 receptor agonist employed in the management of diabetes. Our objective is to provide valuable insights within the framework of ongoing postmarketing safety surveillance for this emerging class of pharmaceutical agents. Furthermore, we provide a concise overview of the efficacy and safety of tirzepatide and discuss its potential application in managing T2DM.

## Case presentation

**Figure 1:**
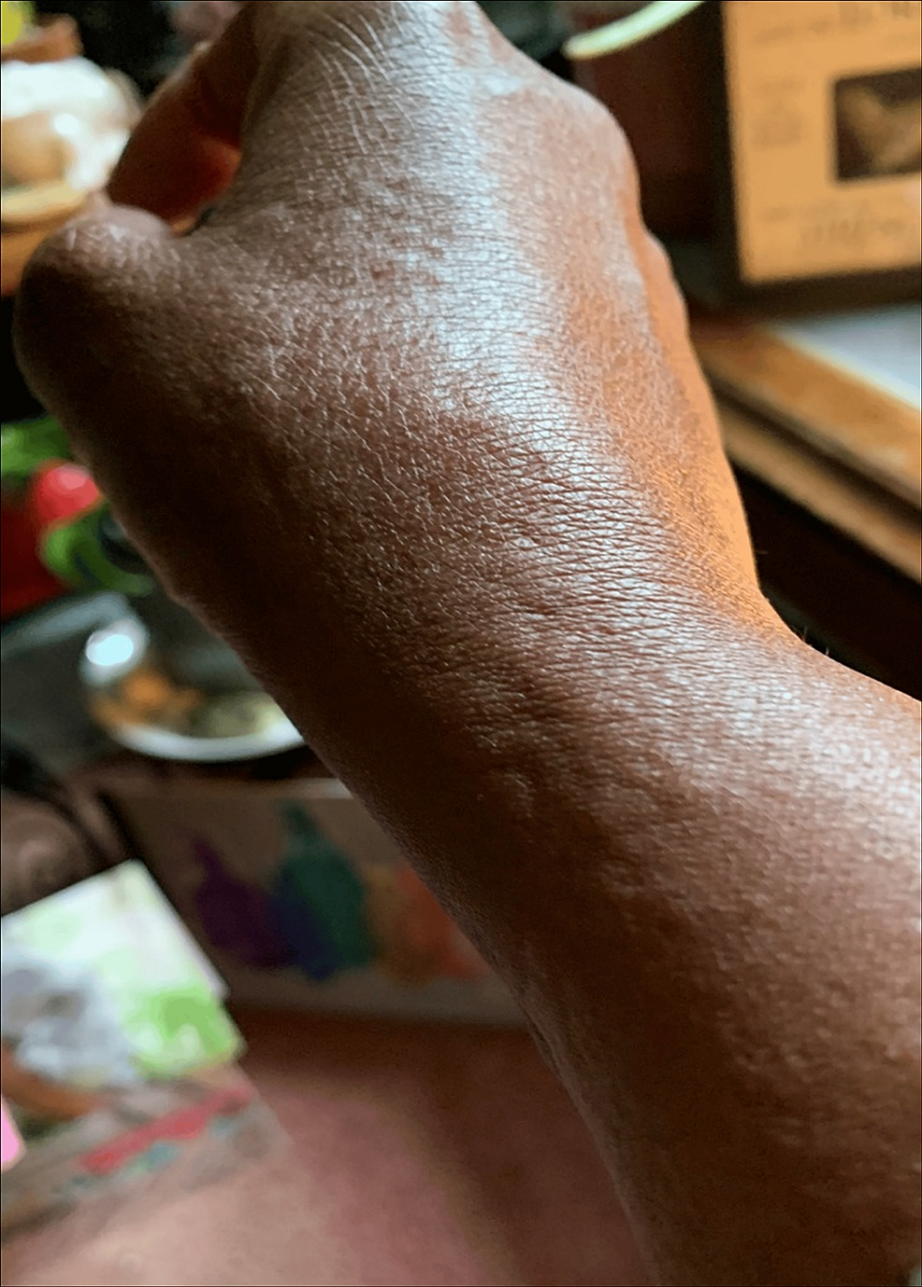
Urticaria on the left hand

**Figure 2:**
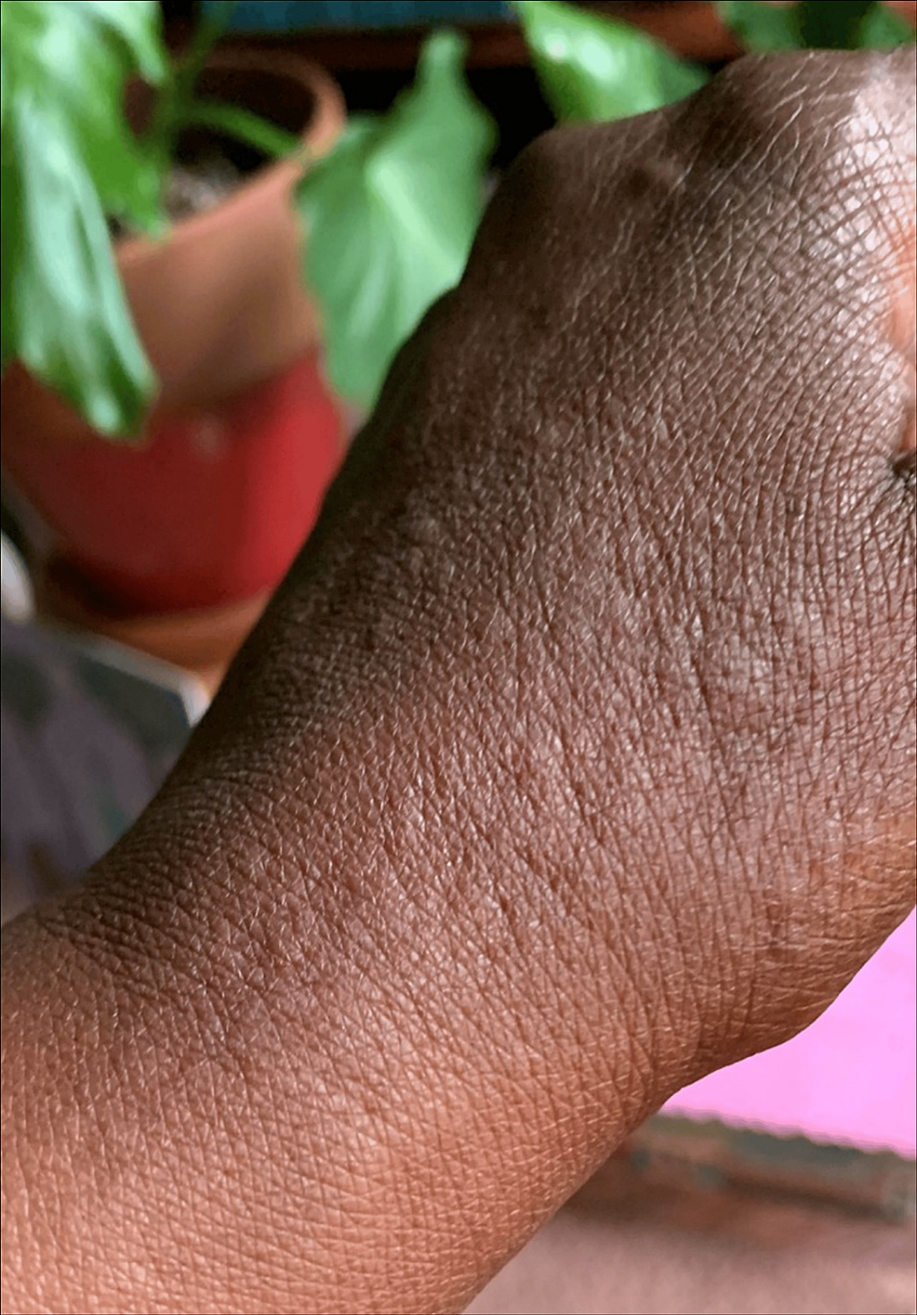
Urticaria on the right hand

**Figure 3:**
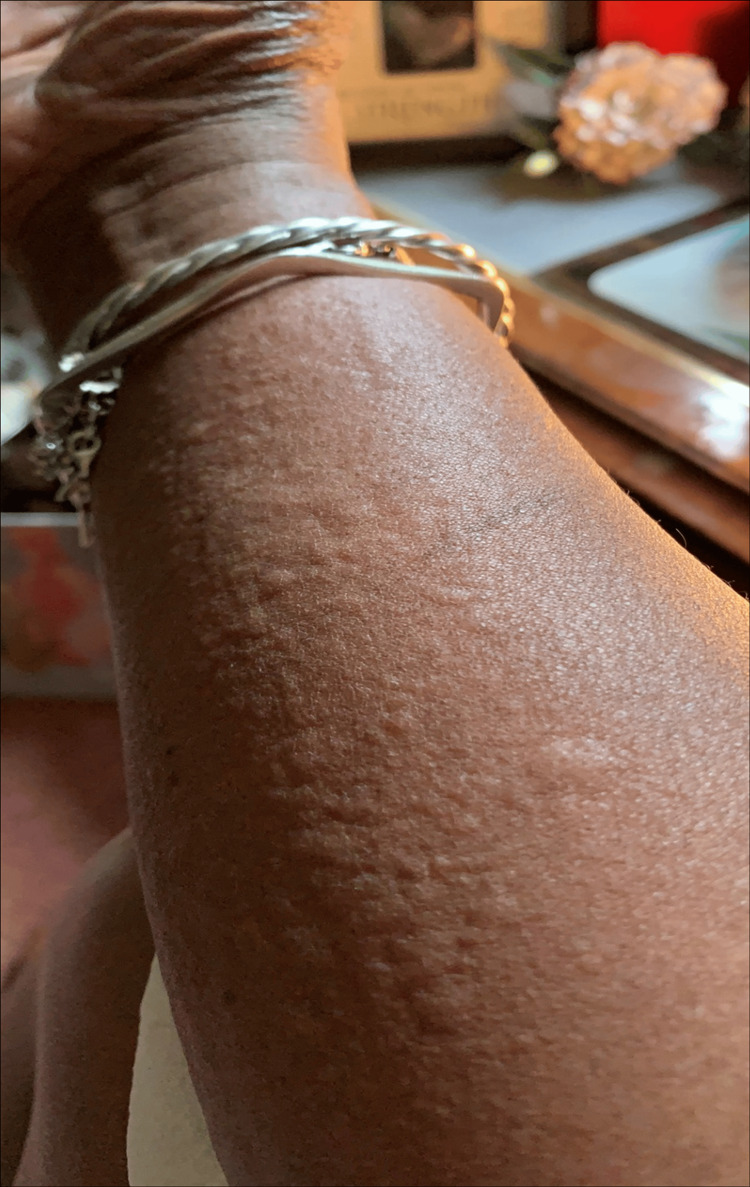
Urticarial rash presented across the arm

A 67-year-old woman from the United States had a personal history of type 2 diabetes, accompanied by several comorbidities, including obesity, hypercholesterolemia, and hypertension. She was receiving basal insulin glargine at a dosage of 38 units at bedtime and metformin 1000 mg twice daily. However, owing to suboptimal glycemic control with the current therapy, in which the HbA1c at that time was 8.2%, and a desire for weight reduction, the patient was prescribed semaglutide as an additional therapy. Facing challenges with her insurance coverage and financial constraints, she discontinued semaglutide after approximately three to four months of usage. Consequently, her physician recommended tirzepatide as an alternative treatment. Around 10-15 minutes after the initial administration of tirzepatide, she experienced a sudden onset of severe disseminated pruritus, accompanied by a generalized urticarial rash that spread across her arms, hands, back, and entire body, excluding her face and neck (Figures 1-3). The patient denied any concurrent swelling of the lips or tongue, difficulty swallowing, or shortness of breath. She self-medicated with antihistamines and made a full recovery without seeking further medical assistance. She subsequently discontinued taking tirzepatide, with no recurrence of symptoms observed, and with no alteration in the concurrent medication regimen.

Upon further inquiry into the patient's medical background, it was discovered that she had received vaccinations for both influenza and COVID-19 two weeks prior to receiving tirzepatide, although she denied experiencing any related symptoms subsequent to receiving the vaccine doses. Prior to the aforementioned reactions, neither food was ingested nor any concurrent medication was taken. Regarding the patient's history of allergies, the patient had a documented allergy to penicillin, presented as skin rashes. Furthermore, the patient reported using semaglutide - a GLP-1 agonist - for around three to four months, during which time she exhibited a favorable response without any adverse allergic reactions.

The patient was diagnosed with an immediate-type allergic reaction to tirzepatide, which is believed to be system-wide and potentially mediated by immunoglobulin E (IgE). The adverse event was subsequently reported to the relevant pharmacovigilance entity.

## Discussion

After a meal, hormones known as incretins are released by enteroendocrine cells in the intestinal mucosa. These hormones play a significant role in regulating the majority of postprandial insulin secretion by beta cells. GLP-1 and GIP are esteemed as prominent and widely recognized incretin hormones. The impact of these hormones is commonly referred to as the "incretin effect," which explains why orally ingested glucose results in a more significant insulin response than intravenous administration, despite having comparable plasma concentrations [6].

GLP-1 functions by inducing insulin release and suppressing glucagon secretion in the hyperglycemic and normoglycemic states. GLP-1 exhibits extrapancreatic effects, encompassing the capacity to delay gastric emptying, enhance satiety, and diminish food intake. These multifaceted actions have been associated with notable reductions in body weight and significant declines in HbA1c levels, thereby holding potential for managing T2DM in affected individuals [6]. GIP is also known for stimulating insulin secretion in response to glucose and contributes more to the incretin effect than GLP-1. However, it differs from GLP-1 in its effect on glucagon secretion: it exhibits glucagonotropic characteristics when blood sugar is normal or low and glucagonostatic characteristics when blood sugar is high. Despite previous observations that supraphysiological infusion of GIP did not stimulate insulin production in people with type 2 diabetes, recent research suggests that GLP-1 and GIP work together to produce a synergistic effect that significantly increases insulin and glucagonostatic response. Thus, although previously thought to be ineffective in lowering blood glucose levels, GIP may have the potential as a glucose-lowering therapy when used in combination with GLP-1 [7].

Utilizing the concept of elevating GLP-1 concentrations, GLP-1 receptor agonists (RAs) that are resistant to dipeptidyl peptidase-4 (DPP-4) were introduced as treatment options for T2DM therapy and are recommended early in the treatment protocols according to current guidelines. This is due to its given benefits of weight reduction, improvement in glycemic control, and positive cardio-nephrovascular effects [8]. Basal insulins are frequently administered in type 2 diabetes patients with insufficient glycemic control using oral glucose-lowering medications. While upping the dose of basal insulin typically enhances glycemic control, it can also escalate the likelihood of hypoglycemia and weight gain, which could impede insulin dose intensification in clinical settings. Nevertheless, incorporating GLP-1 RAs alongside basal insulin has demonstrated superior glycemic control, decreased body weight, and a rising risk of hypoglycemia, while concurrently reducing insulin demands [9].

Dual GIP/GLP-1 RAs are a new class of drugs that combine the effects of two incretin hormones or so-called "twincretin," GLP-1 and GIP, to enhance glycemic control in individuals with type 2 diabetes. This approach is based on the concept of the incretin effect and the potential synergistic effects of these hormones. Compared to the GLP-1 receptor agonist, tirzepatide is a novel, subcutaneous, once-weekly dual GIP/GLP-1 RA drug that has been demonstrated to be superior in controlling HbA1c levels and reducing body weight [10]. The SURPASS-5 clinical trial yielded compelling findings, indicating noteworthy enhancements in glycemic control among individuals with inadequately controlled type 2 diabetes who received tirzepatide as an adjunct therapy to insulin glargine, compared to those who received a placebo, following a 40-week treatment period [3].

Regarding the adverse events (AEs) associated with tirzepatide, its adverse drug reaction profile is comparable to that of GLP-1 agonists. The primary AEs of tirzepatide are gastrointestinal (GI), such as nausea, vomiting, and diarrhea. According to a systematic review and meta-analysis of randomized clinical trials, the investigation demonstrated that tirzepatide exhibited dose-dependent efficacy in improving glycemic control and reducing body weight, without an associated rise in hypoglycemic events. However, an elevation in GI AEs was observed [11]. In a recent systematic review encompassing a meta-analysis of clinical trials evaluating the AEs associated with tirzepatide, GI AEs emerged as the most frequently reported AEs, exhibiting a dose-dependent pattern with prevalence rates ranging from 39% to 49%. Nausea and diarrhea emerged as the predominant GI AEs frequently documented in the study, irrespective of the administered dosage. Mild hypoglycemia (blood glucose < 70 mg/dL) was highest with tirzepatide 10 mg dose, with an incidence rate of 22.6%. However, the rates of severe AEs, such as fatal AEs, severe hypoglycemia, acute pancreatitis, cholelithiasis, and cholecystitis, remained notably low (≤1%) across all administered doses [12]. Additionally, based on the findings of this study, the incidence of hypersensitivity reaction was 3.23% (1.92%-4.86%), 3.03% (2.11%-4.12%), and 2.42% (1.48%-3.58%) for patients taking the 5, 10, and 15 mg doses of the medication, respectively. This was in comparison to 3.88% (1.14%-8.17%) of patients who received a placebo treatment. There were no instances of severe hypersensitivity reactions or injection site reactions found [12].

To the current knowledge, tirzepatide aligns with the established safety profile of GLP-1 and exhibits a manageable AE profile, rendering it a prospective cornerstone in the management of T2DM, insulin resistance, and weight loss in the near future. Nonetheless, all GLP-1 RAs have been associated with documented cases of potentially life-threatening hypersensitivity reactions, including anaphylaxis [13]. Besides, since tirzepatide was recently approved for use, it is essential to be vigilant for any allergic reactions to this drug. In clinical studies of tirzepatide, hypersensitivity reactions, including anaphylaxis and angioedema, have been observed. Some of these hypersensitivity reactions were severe [5,14-19]. Allergic and hypersensitivity reactions were observed in 1% to 2% of tirzepatide-treated patients, slightly higher than the 1% incidence in placebo recipients. Importantly, patients with treatment-emergent antidrug antibodies to tirzepatide did not report severe or serious hypersensitivity or injection-site reactions, although detailed data on this were not disclosed [20].

This paper details a rare and significant case, documenting one of the few known instances of a systemic hypersensitivity reaction to tirzepatide. The hypersensitivity reaction occurred notably within about 15 minutes following the initial dose of tirzepatide, highlighting a direct temporal association with the drug administration. After receiving antihistamines, the patient's symptoms resolved and did not recur upon discontinuation of tirzepatide. No alternative causes for the symptoms were evident in her medical history, making a definitive differential diagnosis challenging. Concerning the influenza and coronavirus vaccines received two weeks prior, there is no direct evidence suggesting that these vaccines influenced tirzepatide functionality or contributed to the hypersensitivity reaction.

## Conclusions

The emergence of dual GIP/GLP-1 receptor agonists like tirzepatide brings new considerations for both short-term and long-term treatment risks. Although early trials showed infrequent systemic hypersensitivity reactions, this case suggests a possible link between tirzepatide and IgE-mediated hypersensitivity, potentially leading to severe reactions. Medical practitioners should be cautious of this association when prescribing tirzepatide, and patients should be informed about these risks. Reporting severe adverse events to pharmacovigilance organizations is crucial for ongoing safety surveillance.
